# Bodyweight Assessment of Enamelin Null Mice

**DOI:** 10.1155/2013/246861

**Published:** 2012-12-26

**Authors:** Albert H.-L. Chan, Rangsiyakorn Lertlam, James P. Simmer, Chia-Ning Wang, Jan C. C. Hu

**Affiliations:** ^1^Department of Periodontics and Oral Medicine, University of Michigan School of Dentistry, 1011 North University Avenue, Ann Arbor, MI 48109-1078, USA; ^2^Department of Biologic and Materials Sciences, University of Michigan School of Dentistry, 1210 Eisenhower Place, Ann Arbor, MI 48108, USA; ^3^Department of Biostatistics, University of Michigan School of Public Health, 109 Observatory Street, 1700 SPH I, Ann Arbor, MI 48109-2029, USA

## Abstract

The *Enam* null mice appear to be smaller than wild-type mice, which prompted the hypothesis that enamel defects negatively influence nutritional intake and bodyweight gain (BWG). We compared the BWG of *Enam^−/−^* and wild-type mice from birth (D0) to Day 42 (D42). Wild-type (WT) and *Enam^−/−^* (N) mice were given either hard chow (HC) or soft chow (SC). Four experimental groups were studied: WTHC, WTSC, NHC, and NSC. The mother's bodyweight (DBW) and the average litter bodyweight (ALBW) were obtained from D0 to D21. After D21, the pups were separated from the mother and provided the same type of food. Litter bodyweights were measured until D42. ALBW was compared at 7-day intervals using one-way ANOVA, while the influence of DBW on ALBW was analyzed by mixed-model analyses. The ALBW of *Enam^−/−^* mice maintained on hard chow (NHC) was significantly lower than the two WT groups at D21 and the differences persisted into young adulthood. The ALBW of *Enam^−/−^* mice maintained on soft chow (NSC) trended lower, but was not significantly different than that of the WT groups. We conclude that genotype, which affects enamel integrity, and food hardness influence bodyweight gain in postnatal and young adult mice.

## 1. Introduction

Enamelin is the largest (~200 kDa) but the least abundant (3–5%) of the three major secretary stage enamel matrix proteins. Twelve mutations in the enamelin gene (*ENAM*, 4q13.2) have been published associated with autosomal dominant amelogenesis imperfecta (ADAI) [1–16]. Clinical features of affected teeth showed thin enamel, with either severe or localized hypoplasia. The enamel phenotype is dose-dependent and ranges from small, well-circumscribed enamel pits to enamel agenesis (when both *ENAM* alleles are mutated) [7]. None of these *ENAM* mutation studies reported a phenotype outside the dentition. In mice, mutations in the *Enam* gene have been induced with the mutagen N-ethyl-N-nitrosourea (ENU), and four separate point mutations have been identified: p.Ser55Ile, p.Glu57Gly, a splice donor site in exon 4, and a premature stop codon in exon 8 (p.Gln176*) [17, 18]. Heterozygous mice exhibited rough and pitted enamel while the null mice showed enamel agenesis. Enamelin null mice were generated by replacing the *Enam* coding sequence from the translation initiation site through exon 7 with a *lacZ* reporter gene [19]. The enamel defects were dose-dependent. The enamel layer was completely absent in *Enam* null mice compared to the mild enamel phenotype in the heterozygotes (*Enam* ^+/−^). A thin, highly irregular, easily abraded mineralized crust over the dentin was observed in the *Enam* null mice. The affected teeth showed significant wear and were generally chalky-white. The histologic, morphometric, and protein/mineral analyses demonstrated that enamelin is essential for proper enamel matrix organization and mineralization. Serum calcium, phosphate, alkaline phosphatase, and glucose levels overlapped normal ranges. 

There is considerable evidence that enamelin is an enamel-specific protein. The *lac*Z knock-in in the *Enam* ^−/−^ only detected enamelin expression in ameloblasts. Although not all ages and organs were surveyed, *Enam* tooth-specific expression is consistent with the human and mouse expressed sequence tag EST profiles. The human (Hs.667018) lists only three *ENAM* transcripts per million for healthy (nondental) tissues. The mouse (Mm.8014) lists four *Enam* transcripts per million in embryonic tissue (which has developing teeth), 1414 per million in molars, and zero in all other tissues. Humans with *ENAM* defects only show an enamel phenotype. Among the reports of 16 kindreds with 12 different amelogenesis imperfecta (AI) causing* ENAM* mutations, none revealed a history of systemic problems associated with the genetic condition. Finally, *Enam* is consistently found to be pseudogenized in vertebrates that have lost the ability to make teeth during evolution [20–23]. These findings support the conclusion that *Enam* defects are unlikely to directly cause a significant reduction in animal bodyweight (BW). Perhaps absence of enamel covering the dentin surface causes pain that discourages eating, especially if the food is hard and requires mastication. 

The relationship between poor oral health and lower bodyweight has been observed in human studies. Acs et al. showed that three-year-old children with dental caries and with at least one pulp-involved tooth weighed 1 kg less than the counterparts [24]. A follow-up study reported that there was significant “catch-up” in growth following complete dental rehabilitation of the children who suffered severe dental caries with pulpal involvement. There are several plausible mechanisms for which dental caries may contribute to underweight and poor growth in young children and one of them is pain and discomfort from dental caries reducing nutritional intake because eating is painful [25]. Severe forms of amelogenesis imperfecta are definitely associated with increased dental pain and can lead to behavioral compensations. When both *ENAM* alleles are defective, the chief complaint typically includes dental pain, particularly thermal sensitivity [7, 12]. Anterior open-bites, which are often associated with tongue-thrust and thumb sucking behaviors, are observed with increased frequency in AI patients, regardless of the genetic etiology, including AI caused by *ENAM* [6], *KLK4* [26], *MMP20* [27], *FAM83H* [28], or *AMELX *[29] mutations.

Enamelin null mice have virtually no enamel [19]. Given that genetic (AI) or environmental (caries and pulp involvement) factors can cause pain upon chewing in humans, it is reasonable to deduce that mice with severe enamel defects might experience eating difficulties that reduce BW gain, especially following the transition from milk to chow. If true, soft chow may prove more tolerable than hard chow, given the lack of enamel. The primary aim of this study was to compare the patterns of BW gain in *Enam* null and wild-type mice during the first 6 weeks of postnatal life, when maintained separately on hard or soft chow. As the mothers of the *Enam* null mice also had no enamel covering the crown of their teeth (which could affect their nutrition and the quantity of their milk), a secondary aim was to evaluate whether maternal bodyweight (DBW) correlated with ALBW among the four experimental groups in the preweaning period.

## 2. Materials and Methods

### 2.1. Animal Protocol

All procedures involving animals were reviewed and approved by the UCUCA Committee at the University of Michigan.

### 2.2. Animal Breeding and Bodyweight Measurement

Wild-type (strain C57BL/6) and *Enam* null (strain C57BL/6) females at the age of 4 to 6 months were mated to males with the same *Enam* genotype and genetic background. The *Enam* null and wild-type mice were maintained separately on soft or hard chow throughout the experiment. The chow was LabDiet 5001 (Purina Mills, St. Louis, MO, USA). Four experimental groups were generated: wild-type hard chow (WTHC), wild-type soft chow (WTSC), null hard chow (NHC), and null soft chow (NSC). The soft chow was water-moistened hard chow, and therefore both contained the same nutritional value. Signs of pregnancy were checked once a day following breeding and each pregnant mouse was transferred to a new cage with minimal disturbance. The mother mice were checked twice a day to determine the accurate birth time (D0) for each litter. Each litter's bodyweight was measured daily as a group at the same time of day using a digital analytical balance (Denver Instrument, Denver, CO, USA) from D0 to D42. The measured value for the weight of the litter divided by the number of pups in the litter gave the average litter bodyweight (ALBW). Each litter was housed with the mother from D0 to D21, after which the litter was weaned and separated by gender. From D22 to D42, the mean litter weight of males and females in the same litter was measured separately on a daily basis. When a mouse died, the average bodyweight (BW) of the remaining litter was used. Dam bodyweight (DBW) was measured daily from D0 to D21. The young adult male mice (at the age of 4-5 months) that were not sacrificed for morphologic examination were weighed, and the mean BW was compared among the four groups.

### 2.3. Morphologic Examination

After the BW analyses, mice at the age of 8 weeks were sacrificed by inhalation anesthesia using isoflurane (Sigma, St. Louis, MO, USA) and perfused with ice-cold 4% paraformaldehyde. Mandibles were dissected from the skull, fixed in 4% paraformaldehyde for 24 h, rinsed with phosphate buffered saline, and stored in 70% ethanol-diethyl pyrocarbonate. Mandibles were separated into halves by incision in the symphysis using a no. 11 scalpel blade. The right hemimandibles were photographed at 3x magnification (SMZ1000, Nikon) for morphologic evaluation under a stereomicroscope.

### 2.4. Statistic Analysis

Descriptive analysis of BW (in grams) was presented as the mean ± SD. ALBW on D0 and on subsequent days at an interval of 7 days until D42 was compared among the four experimental groups by one-way ANOVA and the Tukey test for pairwise group comparisons. DBW was compared on D0, D7, D14, and D21 as well as the mean BW of young adult males using the same method. Subsequently, the mixed model (SPSS 16.0) was implemented to assess the association of ALBW with DBM. Other independent variables include day, group (WHC, WSC, NHC, and NSC), litter size, and combinations. “Day” was treated as a categorical factor. All statistical analyses were conducted by consulting the Center for Statistical Consultation and Research, University of Michigan Ann Arbor, MI, USA.

## 3. Results

Four litters in the wild-type hard chow (WTHC) and wild-type soft chow (WTSC) groups, six litters in the null hard chow (NHC) group and five litters in the null soft chow (NSC) group were available for data analyses. One mother in the WTSC group died for an unknown reason on D17. A foster mother of the same genotype nursed her litter until D21, and the data from this litter was included in the analysis. The foster mother was not also nursing her own pups at that time.

**Figure 1:**
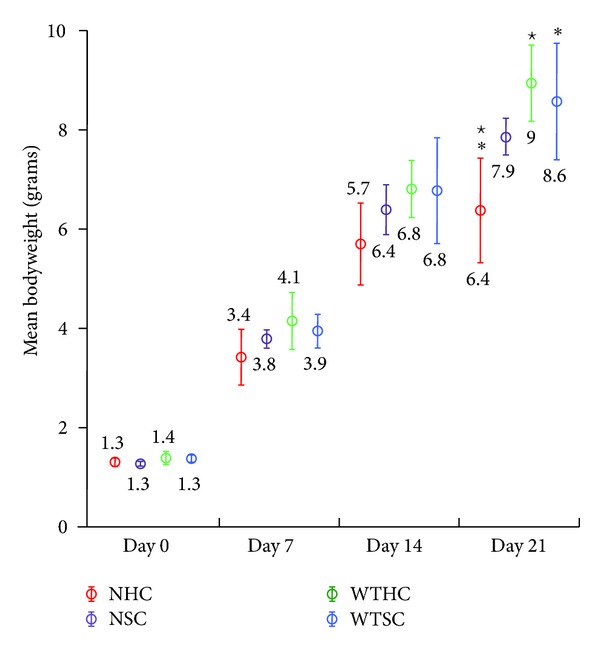
Mean bodyweights of pups at days 0, 7, 14, and 21 (prior to weaning). Four groups of mice were measured and compared wild-type hard chow (WTHC), null hard chow (NHC), wild-type soft chow (WTSC), and null soft chow (NSC). At birth, the mean bodyweight of mice in all groups was similar and showed little variance. Over time, the mean bodyweights showed increasing variance within and between groups. The only statistically significant differences were found at 21 days where the mean bodyweight of null mice provided with hard chow was lower than wild-type mice. Statistically significant differences (SSD) between NHC and WTSC (∗) or WTHC (⋆).

**Figure 2:**
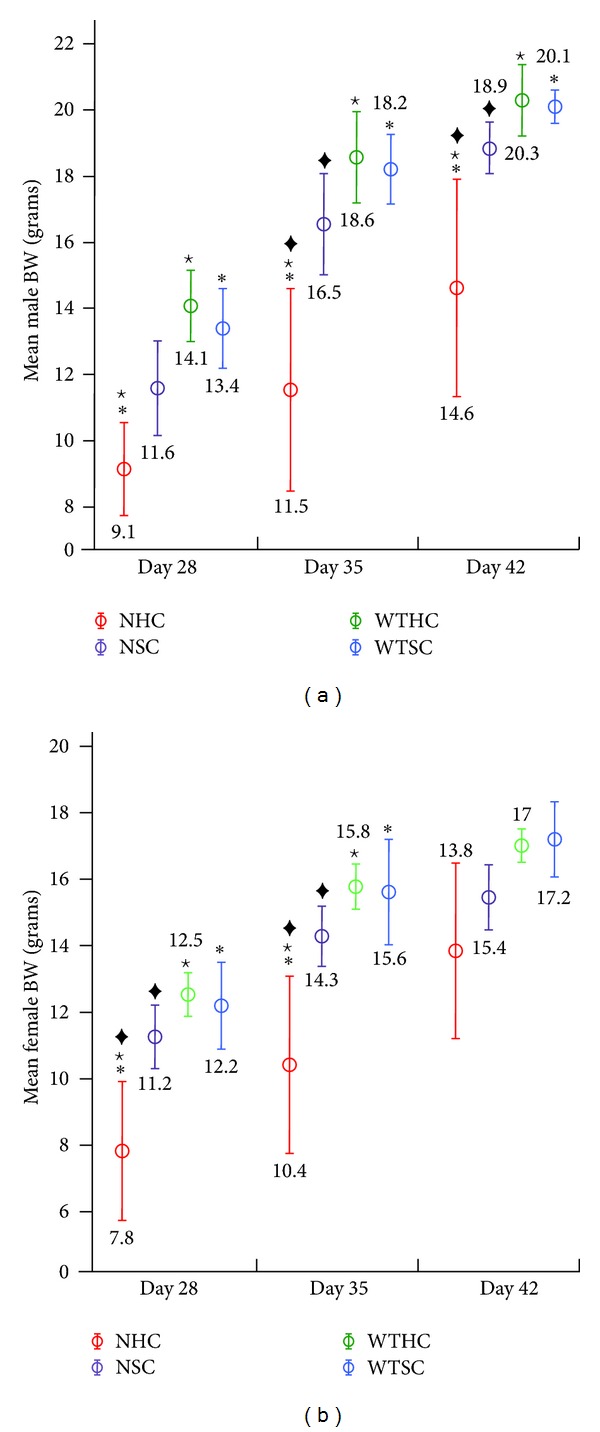
Mean bodyweights of male (a) and female (b) pups after weaning (days 28, 35, and 42). Statistically significant differences (SSD) between NHC and WTSC (∗), WTHC (⋆), or NSC (◆). The null mice on hard chow had a lower mean bodyweight than null mice on soft chow and wild-type mice on hard or soft chow. Although the mean bodyweight of null mice on soft chow tended to have a lower average bodyweight than wild-type mice, the differences were small and not statistically significant. The mean bodyweight of null mice on hard chow varied more than mice in other groups, suggesting differences in how individual mice adapted to eating with defective dentitions.

Plots of the average pup weights (litter weight/pups per litter) ±2 standard errors for each group at specific time points are shown in Figures 1 and 2 and the ANOVA analysis in Table 1. The average bodyweight (BW) of pups from all four groups was not significantly different until D21, when the NHC group was significantly lighter than both of the wild-type groups (Figure 1 and Table 1). In the postweaning period (after D21) when male and female pups were weighed separately, the mean male average litter bodyweight (ALBW) in the NHC group was significantly lower than the other three groups at D35 and D42 (Figure 2(a) and Table 1). The same pattern was observed for female mean ALBW at D28 and D35 (Figure 2(b) and Table 1). These data are consistent with the interpretation that null pups, especially null pups fed hard chow, were gaining weight more slowly because they were eating less due to the lack of enamel on their teeth.

Table 2 shows the mothers' average bodyweights (DBW) of the four groups starting at the birth of their pups (day 0) and again at days 7, 14, and 21. The mothers of the two wild-type groups were significantly heavier than the NSC group on days 0 and 7. On day 14, the mothers of the two null mice groups weighed significantly less than the mothers of the wild-type groups. However, on day 21, no statistically significant differences in maternal bodyweight (DBW) among the four groups were observed, although the null mothers' bodyweights trended lower. Perhaps a larger sample size would have demonstrated significance at day 21 or other variables besides genetic background affected the analysis. The mixed model analyses (Table 3) demonstrated that ALBW was significantly related to the group (*P* = 0.01), litter size (*P* < 0.00), day (*P* < 0.00) but not DBW (*P* = 0.72). We suspect that nursing mothers may exhibit adaptation behaviors in managing dietary intake.

One-way ANOVA analysis of adult male mouse bodyweight and *post hoc* comparison of four experimental groups revealed that the mean bodyweight in the NHC group was still significantly lower than the other three groups (Table 4). Although the average bodyweight of NSC group was 7.8% lower than the average weight of the WTSC, there was no statistical difference between the two groups, which suggests that maintaining *Enam* null mice with tooth defects on soft chow may allow them to obtain adequate nutrition for proper weight gain. 

**Figure 3:**
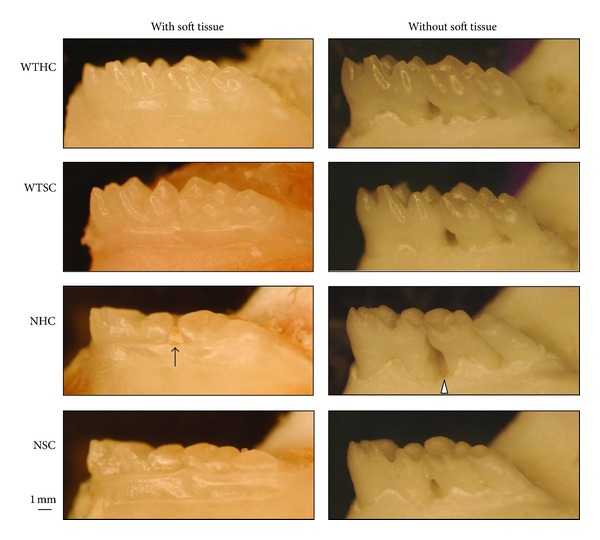
Morphology of mandibular molars at 8 weeks. Normal cuspal morphology, coronal crown contour, and supporting bone structure can be observed among wild-type mice maintained on hard chow (WTHC) or soft chow (WTSC). Flatten cusps and smaller occlusal table are consistent features of null mouse molars (NHC and NSC). Food impaction (arrow) between molars was often observed in the null mice and was associated with bony defects (arrowhead).

Morphologic evaluation of the hemimandibles (Figure 3) showed that the appearance of molars was identical to what was described in previous work [19]. The color of molars was chalky-white and the surface was rough in the null mice. There was no apparent enamel layer, occlusal wear was apparent, and periodontal defects associated food and debris impaction was observed between molars with open interproximal contacts. All findings suggested that the enamel layer of null mice was defective and chewing function may have been compromised.

## 4. Discussion

The ANOVA analyses showed the BW of NHC was significantly different from the two wild-type groups after day 21. In contrast, the BW of NSC was not significantly different from the wild-type groups throughout the 6-week experimental period, even though it was consistently lower than that of wild-type. This result indicated that the food hardness is important for null mice as a factor in determining BW. As soon as the eyes of young mice open, they start to consume solid food [30]. The timing of opening their eyes, around day 14, approximates the timing of BW deviation that we observed. It is plausible that when the young null mice tried to eat solid food as a supplement, they had difficulties because of their defective enamel. Furthermore, they had more trouble gaining weight when they were given only hard chow. The impeded ability to eat hard chow effectively among the null mice resulted in lower bodyweight gain. 

The mean BW of pups decreased by 0.3 gm with each additional pup in the litter on day 21 (the results of the mixed model analyses). The negative relationship between litter size and average litter BW is in accordance with previous studies. The study evaluated the effect of litter size on average pup weight in rats using regression analysis showed that the relation was negative and increased in magnitude from birth to weaning (3 wk) [31]. The effect persisted into the postweaning period; although a compensatory growth spurt seemed to occur during wk 3 to 5. It was noted that when litter size was not adjusted in the mixed model, there was only marginal difference in bodyweight (*P* = 0.08) among groups. The average litter size (average of D0 to D21) in the null groups was about 1 to 2 less than wild-type groups. There were an average of 6.2 pups per litter in null groups, regardless of food type; 7 in WTSC and 8 in WTHC mice. Although no statistic analysis was performed to compare litter size among groups, the fact that the litter size was smaller in null mice suggested that the attrition of pups might have occurred. It may be due to the mother selectively nurse healthier pups and/or smaller/weaker pups in the same litter were outcompeted. Both indicated that the nutrition from null mother mice might not be sufficient compared to the wild-type groups with the same litter size.

It was surprising that there was no correlation between DBW and ALBW. The BW of the null mother mice was consistently lower than that of the wild-type mothers; however, the pattern of litter weight gain did not correspond to that of maternal BW. The ALBW was generally similar among groups until the end of preweaning period. After that time point wild-type pups showed higher BW gain than null pups with soft chow, which in time was heavier than null pups with hard chow. Although not statistically significant, the parallel correlation of DBW and ALBW did not exclude the possibility that the nutritional status of null dams was a factor influencing the observed lower BW of their litters. During the experiment, we found that the values of DBW fluctuated frequently and the magnitude can be as high as 5 grams between two consecutive days. The high BW variation of the individual dam may have contributed toward the finding of no significant difference on ALBW among the experimental groups. One study evaluated the genetic and maternal effects of mice using the cross-fostering technique [32]. Two females littering within a 12-hour period were paired and a random half of pups of each sex were then reciprocally switched within the pair. The BW was taken every 3 days from birth to 6 weeks and weekly weight thereafter until 84 days. The variance due to nurse dams peaked at 12 days, accounting for about 70% of the phenotype variance in D12 weights.

The growth and development of mice in various developmental stages and how they were bred has been studied extensively [30]. In standard husbandry of mice, pups are kept with their mother for the first three weeks after birth, during which their nutrition primarily comes from mother's milk. Young mice start to consume solid food as soon as their eyes open roughly around day 14. They are weaned on day 21, signaling the end of their dependence on their mother. At the age of 6 weeks, they are considered young adult and can be active in reproduction. In a study assessing the impact of inherited enamel defects on bodyweight gain of amelogenin (*Amelx*) null mice [33], it was reported that the average BW of the null mice was less than that of the wild-type mice on each day of the 3-week preweaning period. It is possible that the result is due partially to decreased nutritional intake starting from the affected mother mice. It would be interesting to determine if the BW difference would be even more significant after the young mice start to eat with their defective teeth independently from their mother. 

Recently, a phenotyping screening of two enamelin-mutant mouse lines originating from an ENU mutagenesis project for dominant mutations on a C3HeB/FeJ genetic background observed dominant effects of heterozygous enamelin mutations on bone and energy metabolism, as well as on clinical chemistry and hematological parameters, suggesting that enamelin plays a critical role in other organs besides developing teeth [34]. The first mouse, with the p.Gln176* mutation, had previously been characterized without noting systemic effects [18]. The second mouse *Enam* mutation (c.A382T; p.Lys128*) was dominant so that mice heterozygous and homozygous for the mutation both showed the same level of defective “whitish” enamel. They concluded that the heterozygous enamelin truncations likely resulted in defective energy metabolism possibly from subtle changes in liver and/or pancreas function. This conclusion is hard to reconcile with the fact that not a single enamelin transcript is listed among the 111,391 (mouse) or 205,232 (human) ESTs for liver and 106,259 (mouse) or 213,410 (human) ESTs for pancreas. Enamelin is a member of the secretory calcium-binding phosphoproteins (SCPPs) encoded by a family of genes clustered on chromosome 4q13 in humans and chromosome 5 in mice [35, 36] that are critical for many processes, such as bone and tooth biomineralization, saliva, and lactation. Perhaps, there are undetected mutations affecting these linked genes that account for their findings. The results of this study cast doubt upon the claim that their mice with severe enamel defects ate as much hard chow as wild-type mice. If there are no differences in food intake by mice with and without enamel, what is the selective pressure that maintains the enamel layer during evolution?

In this study, we first tested the hypothesis that *Enam* null mice have lower bodyweights than wild-type mice. Our results showed that *Enam* null mice have significantly lower bodyweights than wild-type mice starting at day 21, when the mice are weaning. We also showed that weaned null mice fed on hard chow tend to have lower bodyweights than null mice fed on soft chow, revealing that at least part of the lower bodyweight can be explained by eating difficulties secondary to the lack of enamel on the dentition. Our results, in conjunction with evidence that enamelin is expressed specifically by ameloblasts and there is an absence of selection pressure to maintain *Enam* in vertebrates that have lost the ability to make enamel during evolution, support the conclusion that enamelin does not perform necessary functions outside of dental enamel formation.

## Figures and Tables

**Table 1:** One-way ANOVA analyses of ALBW at days 0, 7, 14, 21, 28, 35, and 42. The average litter bodyweights (g) with standard deviations in parentheses are presented. Each litter weight was divided by the number of pups in the litter. Statistically significant (*P* < 0.05) differences between groups within each time point are noted.

Day	Gender	WTHC	WTSC	NHC	NSC
0	M + F	1.4 (0.1)	1.4 (0.1)	1.3 (0.1)	1.3 (0.1)
7	M + F	4.1 (0.5)	3.9 (0.4)	3.4 (0.3)	3.8 (0.2)
14	M + F	6.8 (0.6)	6.8 (1.0)	5.7 (0.8)	6.4 (0.6)
21	M + F	9.0^⋆^ (0.8)	8.6* (1.2)	6.4^⋆∗^ (1.2)	7.9 (0.4)
28	M	14.1^⋆^ (1.1)	13.4* (1.3)	9.1^⋆∗^ (1.8)	11.6 (1.6)
	F	12.5^⋆^ (0.7)	12.2* (1.3)	7.8^⋆∗‡^ (2.1)	11.2^‡^ (1.1)
35	M	18.6^⋆^ (1.4)	18.2* (1.1)	11.5^⋆∗‡^ (3.8)	16.5^‡^ (1.7)
	F	15.8^⋆^ (0.7)	15.6* (1.6)	10.4^⋆∗‡^ (2.7)	14.3^‡^ (1.0)
42	M	20.3^⋆^ (1.1)	20.1* (0.5)	14.6^⋆∗‡^ (3.7)	18.9^‡^ (0.9)
	F	17.0 (0.5)	17.2 (1.2)	13.8 (2.3)	15.4 (1.2)

Statistically significant differences (SSDs) between NHC and WTHC (^*⋆*^); NHC and WTSC (*); NHC and NSC (^‡^). Until day 21, the males and females of each litter were weighed together. There were no statistically significant differences among the groups until day 21, suggesting that bodyweight differences might be associated with the transition to eating chow.

**Table 2:** One-way ANOVA analyses of dam bodyweights on litter days 0, 7, 14, and 21. The average bodyweights (g) of the mothers with standard deviations in parentheses are presented. Statistically significant (*P* < 0.05) differences between groups within each time point are noted.

Group	WTHC	WTSC	NHC	NSC
*N *	4	4	6	5
D0	31.94^†⋆^(0.84)	30.27^*||*^ (1.74)	27.92^⋆^(1.37)	26.48^†*||*^ (1.59)
D7	35.33^†⋆^(1.70)	33.77^*||*^ (1.53)	30.74^⋆^(1.80)	29.61^†*||*^ (2.33)
D14	36.56^†⋆^(3.54)	36.68^∗*||*^ (2.59)	31.73^⋆∗^ (2.18)	30.89^†*||*^ (1.99)
D21	34.00 (4.77)	34.23 (4.71)	31.38 (2.52)	31.12 (2.15)

Statistically significant differences (SSD) between NHC and WTHC (^*⋆*^); NHC and WTSC (*); NSC and WTHC (^†^); NSC and WTSC (^*||*^); *N*: number of litters. This data shows that the null mothers fed hard chow did not show significant differences in bodyweight with null mothers fed soft chow. The null mothers' bodyweights were smaller than those of the wild-type mothers.

**Table 3:** Mixed-model analyses of independent variables and ALBW. The potential for independent variables to influence the average litter bodyweight was assessed. The intercept is the ALBW at D21. A *P* value <0.05 was accepted as significant.

Source	Numerator df	Denominator df	*F*	*P*
Intercept	1	87.32	405.12	<0.00
Day	21	108.72	14.67	<0.00
Group	3	16.04	5.45	0.01
Litter size	1	74.81	22.00	<0.00
DBW	1	225.85	0.13	0.72
Day-group	63	103.78	1.70	0.01
Day-litter size	21	109.79	3.51	<0.00
Day-DBW	21	110.17	0.85	0.66

df: degrees of freedom. The table shows that factors such as litter size could influence the average litter bodyweight, while the mother's bodyweight (DBW) did not.

**Table 4:** One-way ANOVA analyses comparing adult male mouse bodyweights. Statistically significant (*P* < 0.05) differences between groups at time point of 4-5 months are noted.

Group	WTHC	WTSC	NHC	NSC
*N*	9	13	10	11
Mean BW (g)	25.64^⋆^ (1.55)	28.03* (1.08)	21.94^⋆∗‡^ (4.54)	25.85^‡^ (2.09)

Statistically significant differences (SSD) between NHC and WTHC (^*⋆*^); NHC and WTSC (*); NHC and NSC (^‡^). The bodyweight of NHC adult male mice was significantly lower than that of the other three groups.
